# Repair of Temporal Bone Encephalocele following Canal Wall Down Mastoidectomy

**DOI:** 10.1155/2014/271824

**Published:** 2014-09-22

**Authors:** Sarantis Blioskas, Ioannis Magras, Stavros Polyzoidis, Konstantinos Kouskouras, Georgios Psillas, Stamatia Dova, Konstantinos Markou

**Affiliations:** ^1^1st Department of Otorhinolaryngology-Head and Neck Surgery, AHEPA Hospital, Aristotle University of Thessaloniki, 1 Stilponos Kyriakidi Street, 54636 Thessaloniki, Greece; ^2^1st Department of Neurosurgery, AHEPA Hospital, Aristotle University of Thessaloniki, 1 Stilponos Kyriakidi Street, 54636 Thessaloniki, Greece; ^3^Department of Radiology, AHEPA Hospital, Aristotle University of Thessaloniki, 1 Stilponos Kyriakidi Street, 54636 Thessaloniki, Greece

## Abstract

We report a rare case of a temporal bone encephalocele after a canal wall down mastoidectomy performed to treat chronic otitis media with cholesteatoma. The patient was treated successfully via an intracranial approach. An enhanced layer-by-layer repair of the encephalocele and skull base deficit was achieved from intradurally to extradurally, using temporalis fascia, nasal septum cartilage, and artificial dural graft. After a 22-month follow-up period the patient remains symptom free and no recurrence is noted.

## 1. Introduction

Impaction of cerebral tissue into the temporal bone (mastoid cavity and/or middle ear) is known as temporal encephalocele. The condition was first documented as early as 1902 by Caboche [1]. A congenital or acquired bony defect of the tegmen is a consistent prerequisite for the development of this rare, yet potentially lethal condition. The etiology of such a defect can be attributed to chronic otitis media with or without cholesteatoma, temporal trauma, otologic surgery, neoplasia, irradiation, congenital cranial base defects, or idiopathic causes [2–4].

Iatrogenic etiology was very common during the early part of the twentieth century and prior to modern antibiotic era, since trephination and draining through the infected mastoid and dura were the mainstay management of brain abscesses, after otitis media. However, encephalocele after mastoid surgery remains today a rare, yet existing occurrence.

We report a rare case of a temporal bone encephalocele after a canal wall down mastoidectomy performed to treat chronic otitis media with cholesteatoma. The patient was treated successfully through an intracranial approach.

## 2. Case Report

A 36-year-old male was referred to the Otolaryngology-Head Neck Surgery Department, due to a right sided chronic otitis media with cholesteatoma. The patient underwent a T-tube placement 4 years prior to referral, to treat chronic otitis media with effusion and tube placement resulted to a residual tympanic perforation and cholesteatoma.

After a thorough clinical and radiological evaluation a typical canal wall down mastoidectomy was performed. Cholesteatoma debris was meticulously removed along with granulation tissue and infected mucosa. Only an egg cell bone was left intact at the tegmen area and although careful drilling removed all mucosa, no apparent iatrogenic dural injury was noted. Yet part of the roof of the external auditory meatus appeared intraoperatively to be eroded by infection and a “fragile” dura could not be surely excluded.

**Figure 1:**
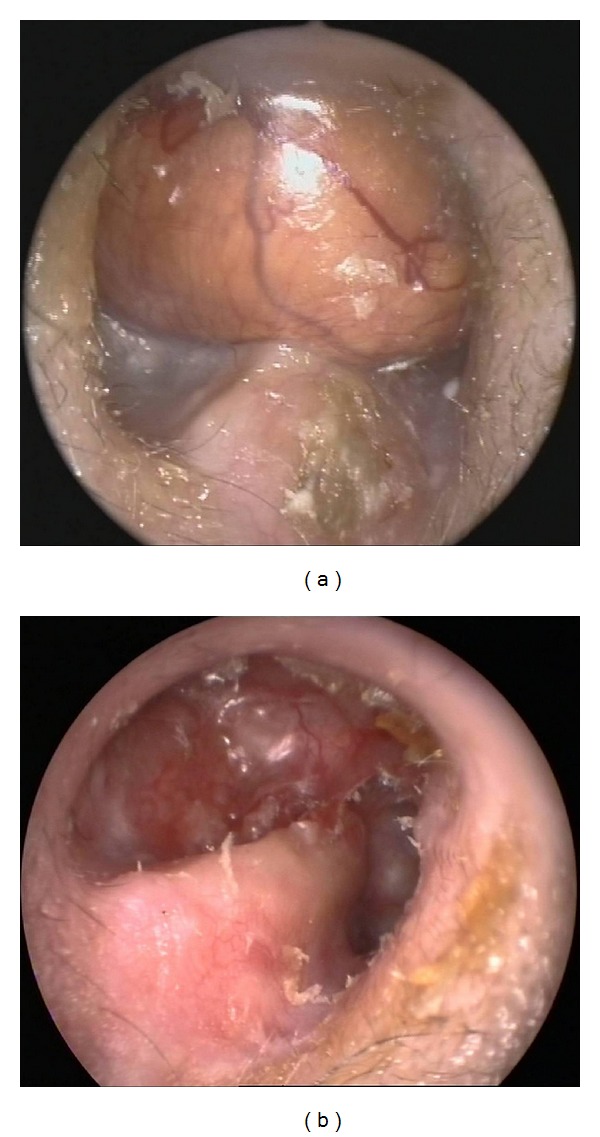
(a) Otoscopy reveals an opalescent pulsating mass in the external auditory canal. (b) Otoscopy after encephalocele repair shows no signs of recurrence.

**Figure 2:**
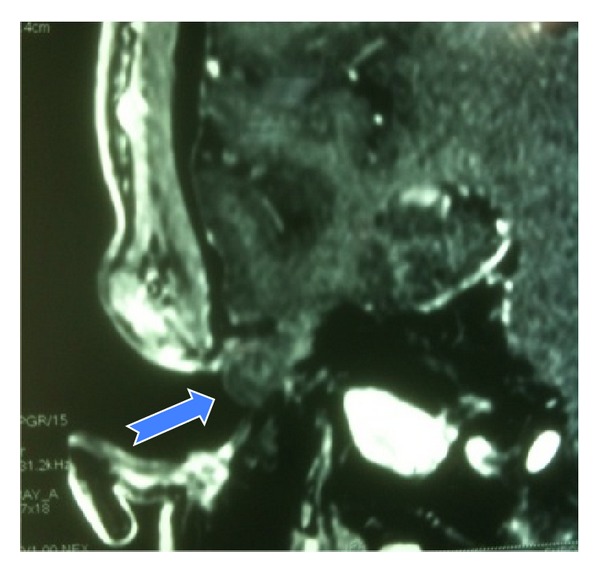
Preoperative imaging; T1 weighted contrast-enhanced MRI (coronal view) revealing the temporal bone encephalocele (blue arrow).

On typical follow-up after 12 months otoscopy revealed an opalescent mass protruding from the roof of the external auditory canal (Figure 1(a)). Imaging confirmed this lesion to be a temporal bone encephalocele (Figure 2). A multidisciplinary approach including both an otologic and a neurosurgical team was decided and informed consent was obtained to surgically treat the lesion.

**Figure 3:**
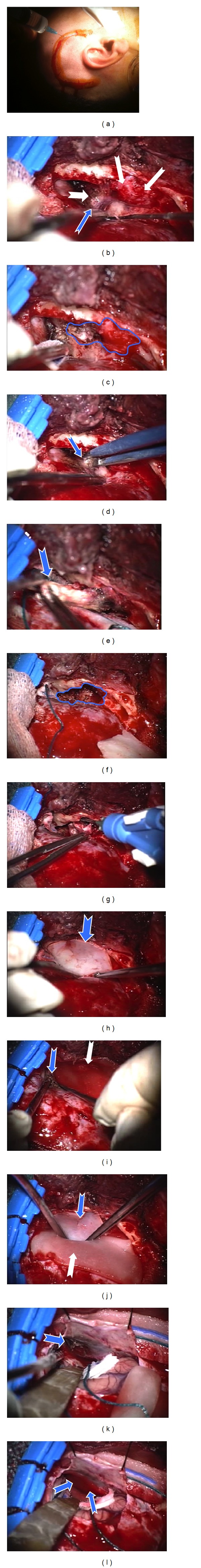
(a) Preparation for the semilunar skin incision immediately over the right ear. (b) Encephalocele: dural gap (blue arrow) and protruding brain (white arrows). (c) Bony deficit of elliptical shape with abnormal borders on the roof of the right external auditory meatus (blue borderline, prior to removal of the protruding brain from its cavity). (d, e) Cauterization and resection of the encephalocele (blue arrows). (f) Bony deficit after removal of the protruding brain from its cavity (blue borderline). (g) Extracranial reconstruction of the bony deficit with use of the temporalis fascia graft and glue. (h) Intracranial reconstruction of the bony deficit with the nasal cartilage. (i) Dural gap extradural view (blue arrow: dural gap, white arrow: artificial dura graft). (j) Artificial dural graft was used to cover the dural gap extradurally (in touch on one side with the dura and on the other with the cartilage) (blue arrow: nasal cartilage, white arrow: artificial dura graft). (k) Dural gap intracranial view (blue arrow). (l) Artificial dura graft to cover dural gap intracranial view (blue arrows).

Patient was placed on the supine position and the operation was completed in three sequential stages under general anaesthesia. The first stage consisted of the preparation for the autograft of the cartilaginous nasal septum. The second consisted of the repair of the encephalocele and the reconstruction of the skull base and was completed in two substages, one extradural and one intradural. Patient's head was rotated 30° towards the left side and the skin was incised in a semilunar fashion immediately over the right ear (Figure 3(a)). A temporalis fascia autograft was prepared, resected, and preserved to be used at a later stage of the operation and a craniotomy of the same fashion followed. The encephalocele was initially approached intracranially-extradurally. Temporal lobe brain tissue was found protruding through a circular shape deficit of the skull base dura (Figure 3(b)) and then through a bony deficit of elliptical shape with abnormal borders on the roof of the external auditory meatus (Figure 3(c)) covered only by epidermis (being ~1.7 cm long in its biggest dimension). The protruding brain was cauterized and resected (Figures 3(d) and 3(e)) and the cavity where the brain was protruding was revealed (Figure 3(f)). The skull base deficit was reconstructed firstly with use of a temporalis fascia autograft, which was placed extracranially into the cavity that was occupied by the encephalocele, tangentially to the skull base deficit, attached to the surrounding tissues and fixed with tissue glue (Figure 3(g)). Secondary, the cartilaginous nasal septum was placed intracranially at the same site (Figure 3(h)). An artificial dural graft (Duragen size 5.0 cm × 5.0 cm) was used to cover the dural gap extradurally being in touch on one side with the dura and on the other side with the cartilage (Figures 3(i) and 3(j)). The next substage was performed completely intradurally. The dura was incised in a semilunar fashion and the dural deficit was identified. Another artificial dural graft (Duragen size 2.5 cm × 2.5 cm) was used to cover the dural gap intradurally (Figures 3(k) and 3(l)) being in touch on one side with the brain and on the other side with the dura. Thus, an enhanced layer-by-layer repair of the encephalocele and skull base deficit was achieved from intradurally to extradurally. The third stage of the operation consisted of the packing of the external auditory meatus. Postoperative course was uneventful and the patient was discharged the fifth postoperative day following a CT scan, which confirmed the reconstruction of the scull base and the repair of the encephalocele.

After a 22-month follow-up the patient remains symptom free and no recurrence is noted (Figure 1(b)).

## 3. Discussion

Temporal bone encephalocele is a rare entity that occurs mostly as a complication of a middle ear infection, especially with cholesteatoma, or as a complication of surgical intervention aimed at the eradication of that disease. It is important to remember that a bony defect of the skull base alone does not necessary lead to cerebral protrusion because the dura mater is structurally strong and capable of supporting the brain even when large bone defects occur during otologic surgery. It appears that dural injury rather than simple exposure is a prerequisite for iatrogenic encephalocele [5].

Diagnosis of temporal bone encephalocele may be particularly obscure and challenging due to the nonspecificity of findings. Clinical symptomatology may encompass conductive or mixed hearing loss, cerebrospinal fluid (CSF) otorrhea and rarely seizure disorders, recurrent meningitis or aphasia. The typical otoscopic finding is the presence of an opalescent pulsating mass in the external auditory canal or in the mastoid cavity. Yet definitive diagnosis may be ultimately based on radiographic or even intraoperative findings.

Radiological evaluation is useful for diagnosing temporal bone encephalocele. High-resolution computed tomography (HRCT) and magnetic resonance imaging (MRI) are the preferred means in establishing the diagnosis and excluding other potential congenital communications or otologic infection. The location, number, and size of the bony defects are well demonstrated with HRCT. Direct coronal slices with bone algorithm are required to reliably reveal such bone defects. Yet computed tomography (CT) is nonspecific in the diagnosis of encephalocele since it has a limited ability to resolve subtle distinctions in density between cerebral tissue, cholesteatoma, cholesterol granuloma granulation tissue, or other soft-tissue masses inside the middle ear cavity. MRI, with its affinity for soft-tissue definition, is the ideal method to differentiate these conditions and complements HRCT study in patients suffering from encephalocele.

Surgical correction remains the treatment of choice, since complications of encephalocele such as meningitis or a brain abscess are fatal. Surgical management of temporal lobe protrusion goes back to 1913, when Canfield [6] reported the closure of dural defect with fascial repair. Today various reconstruction techniques are described in the literature, yet they all require one of the four approaches available: transmastoid, intracranial, combined intracranial/mastoid and middle ear obliteration [2]. Each approach has specific advantages and final choice is ultimately dictated by factors like the position and size of the defect on preoperative imaging, the etiology of encephalocele, and the preoperative audiometry results. However, the type of the approach can be modified by intraoperative findings like the presence of chronic infection in the middle ear or intraoperative active CSF leakage [7]. In our case an intracranial approach was adopted since such an approach facilitated maximal exposure of the defect and allowed optimum surgical manipulation, considering a radical mastoidectomy preceded and a subsequent large cavity was established.

Furthermore restoration of dural integrity is imperative in successful management of encephalocele. Careful dural closure and firm support of intracranial contents are of crucial importance to prevent recurrence. Different materials can be used to repair both the bone and dural layer. Autogenous graft material is preferred and temporalis fascia remains the dominant choice for use as dural graft because of its accessibility. Yet simple repair with only a single layer of temporalis fascia is associated with a high rate of recurrence [7]; thus rigid tissue like muscle, cartilage, or bone in a multilayer repair to cover the bone defect is critical. Perichondrial-auricular cartilage graft, septal cartilage wrapped with temporalis fascia, and cranial bone sandwiched with temporalis fascia have been described [3]. Intradural graft placement is generally preferred as it guarantees that protruded and possibly infected parts of cortical contents are not pushed intracranially. Such a graft placement also facilitates better resistant to intracranial pressure. Despite these facts extradural graft placement has also been described [8]. Herniated cerebral tissue is generally regarded devitalized, nonfunctional and potentially infected. It is compromised viability results from strangulation and ischemia, yet its nonfunctionality remains the case, despite clinical signs or appearance. Thus it is advocated that herniated tissue should be amputated. Such an excision seems to have no neurological complication. Nevertheless few have cautioned against this practice due to potential risk of temporal lobe aphasia or seizure and suggest the reduction of herniated tissue intracranially when it seems to be viable [7, 8].

In our case an enhanced layer-by-layer repair of the encephalocele and skull base deficit was achieved from intradurally to extradurally, using temporalis fascia, nasal septum cartilage, and artificial dural graft. To our opinion, such a technique ensures adequately firm closure and prevents recurrence. Protruded cerebral tissue was cauterized and resected according to literature common practice and no postoperative neurological deficits were noted.

## 4. Conclusion

We report the case of a temporal bone encephalocele after mastoidectomy performed to treat chronic otitis media with cholesteatoma. The patient was successfully treated via an intracranial approach and no recurrence has been noted after 22 months of follow-up.
